# Disability and participation in breast and bowel cancer screening in England: a large prospective study

**DOI:** 10.1038/bjc.2017.331

**Published:** 2017-10-03

**Authors:** S Floud, I Barnes, M Verfürden, H Kuper, T Gathani, R G Blanks, R Alison, J Patnick, V Beral, J Green, G K Reeves

**Affiliations:** 1 Cancer Epidemiology Unit, Nuffield Department of Population Health, University of Oxford, Roosevelt Drive, Oxford OX3 7LF, UK; 2 UCL Great Ormond Street Institute of Child Health, 30 Guilford Street, London WC1N 1EH, UK; 3 International Centre for Evidence in Disability, London School of Hygiene & Tropical Medicine, Keppel Street, London WC1E 7HT, UK; 4 Oxford University Hospitals NHS Foundation Trust, John Radcliffe Hospital, Headley Way, Headington, Oxford OX3 9DU, UK

**Keywords:** disability, screening, participation, bowel cancer, breast cancer

## Abstract

**Background:**

There is limited information about participation in organised population-wide screening programmes by people with disabilities.

**Methods:**

Data from the National Health Service routine screening programmes in England were linked to information on disability reported by the Million Women Study cohort participants.

**Results:**

Of the 473 185 women offered routine breast or bowel cancer screening, 23% reported some disability. Women with disabilities were less likely than other women to participate in breast cancer screening (RR=0.64, 95% CI: 0.62–0.65) and in bowel cancer screening (RR=0.75, 0.73–0.76). Difficulties with self-care or vision were associated with the greatest reduction in screening participation.

**Conclusion:**

Participation in routine cancer screening programmes in England is reduced in people with disabilities and participation varies by type of disability.

People with disabilities may be less likely to participate in cancer screening than people without disabilities (Andresen *et al*, 2013), but evidence is lacking for England where all people in the relevant age ranges are routinely invited for free cancer screening, regardless of health status or ability to pay. Given that the UK 2010 Equality Act requires equitable access to all National Health Service (NHS) screening programmes, our aim was to investigate disparities in participation in breast and bowel screening related to specific types of disability in a large prospective cohort of women in England. We also considered whether not having a partner or not having access to a car would further reduce participation in breast screening given that it involves tests outside the home.

## Methods

A total of 1.2 million women in England, aged 56.2 years on average, were recruited to the Million Women Study in 1996–2001. Participants completed a questionnaire on socio-demographic, lifestyle, and reproductive factors and were sent postal re-survey questionnaires at 3–5 yearly intervals. The study design has been described previously (Million Women Study Collaborators, 2003). Questionnaires and data access policies can be viewed online at http://www.millionwomenstudy.org. All participants gave written consent to follow-up and the study has ethical approval (REC 97/5/001). Participants were linked by their unique NHS number to routinely collected NHS databases, through which they are followed up for death, emigration or cancer registration. Participants were also linked to NHS cancer screening records.

In 2006–2007, Million Women Study participants were asked for the first time about disability on the 8-year re-survey questionnaire, and so this re-survey forms the baseline for these analyses. Women were considered to have a mobility disability if they answered ‘yes’ to the question ‘Do you have difficulty walking up a flight of stairs?’ and reported their walking pace to be ‘slow’. Women were considered to have a hearing, vision, or memory disability if they reported their hearing, or eyesight (with glasses if worn), or memory to be poor (from a four-point scale: ‘excellent’ ‘good’ ‘fair’ ‘poor’). If women agreed that they had difficulty bathing or dressing themselves, then they were deemed to have a self-care disability. Women were asked if they received non-means-tested disability benefits or disabled parking benefits (‘disability living allowance, attendance allowance, or blue badge’).

In the time period under study (2006–2011), routine breast screening was offered to all women aged 50–70 years every 3 years (Department of Health, 2000). Routine bowel screening was offered to all men and women aged 60–69 years from 2006 (extended to 74 years from 2010) every 2 years by sending them a faecal occult blood test kit in the post (NHS, 2007; Blanks *et al*, 2015).

### Statistical analysis

Women who responded to the 8-year re-survey and who were subsequently invited for screening were eligible for this analysis. After excluding 16 489 women with missing information on all the disability variables, and 50 427 with previous cancer (except non-melanoma skin cancer), there remained 445 579 women routinely invited for breast screening and 449 058 routinely invited for bowel screening. Separate analyses were conducted for breast and bowel screening.

Using logistic regression, we calculated odds ratios (referred to as relative risks (RRs)) and 95% confidence intervals (CIs) for participation in screening, comparing women with any disability to women with no disabilities. All analyses were adjusted for age at baseline (54–59, 60–64, 65–69, 70+ years), region (nine cancer registration regions), area deprivation (quintiles calculated at recruitment, based on the Townsend Index, a score incorporating census area data for employment, car ownership, home ownership, and household overcrowding; Townsend *et al*, 1988), ethnicity (white, black, Asian, other), educational qualifications (tertiary, secondary, technical, none but completed compulsory schooling, none and did not complete compulsory schooling), marital status (partnered, not partnered), car availability, body mass index (<18.5, 18.5–24.9, 25–29.9, ⩾30 kg m^−2^), and smoking (never, past, current). Missing values of the adjustment variables were assigned to a separate category (<2% missing for every adjustment variable, except for body mass index and ethnicity: 6% and 16% missing, respectively). In a sensitivity analysis, we restricted the sample to women with complete information on all variables.

We compared the RRs for participation in screening in groups of women defined by car availability and marital status.

Analyses used STATA 14 (StataCorp., College Station, TX, USA).

## Results

Overall, 23% of women invited to either breast or bowel screening reported having a disability and mobility disability was the most common type (18%) (Supplementary Table S1). Women with disabilities were on average older, more deprived, and had fewer educational qualifications than women with no disabilities (Table 1). They were less likely to be married or have a car available and more likely to smoke and be obese. Characteristics varied somewhat by type of disability, for example, women with vision disabilities or self-care difficulties tended to be more deprived and were less likely to have access to a car, and women with mobility disabilities or self-care difficulties were more likely to be obese (Supplementary Table S2).

**Figure 1:**
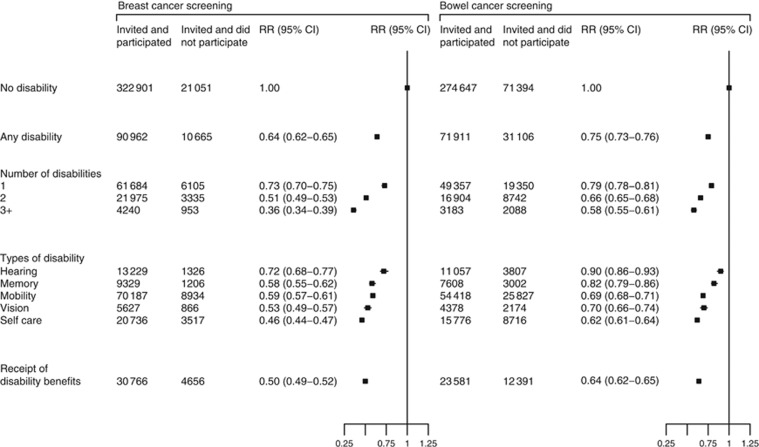
**RR (95% CIs) for participating in breast and bowel cancer screening by various measures of disability.** Any disability includes any type of disability and receipt of disability benefits. RRs are adjusted for age, region, deprivation, ethnicity, marital status, car availability, body mass index, and smoking.

Women with disabilities were less likely to attend breast screening or to complete a bowel screening test than women with no disabilities, even after adjustment for socio-demographic and lifestyle factors (Figure 1). Women with disabilities were 36% less likely to attend breast screening and 25% less likely to participate in bowel screening. For breast screening, the magnitude of disparity was greatest for women with self-care difficulties (RR=0.46, 0.44–0.47) and vision disabilities (RR=0.53, 0.49–0.57). For bowel screening, the magnitude of disparity was greatest for women with self-care difficulties (RR=0.62, 0.61–0.64), mobility disabilities (RR=0.69, 0.68–0.71), and vision disabilities (RR=0.70, 0.66–0.74). Women who reported a greater number of disabilities were even less likely to participate in screening. A sensitivity analysis using only participants with complete information on all covariates produced similar results to the main analysis (Supplementary Figure S1).

Women with any disability who had no access to a car were significantly less likely to participate in breast screening than similar women who had access to a car (Table 2). There was no strong evidence that marital status modified the association between any disability and participation in screening for either breast or bowel cancer.

## Discussion

This study provides large-scale evidence that women in England with disabilities are less likely to participate in free routine screening for breast and bowel cancer than women without disabilities. This is an important finding given the high prevalence of women living with a disability that causes substantial difficulty with day-to-day activities, estimated at 32% in the United Kingdom for women aged 60–64 years (Department for Work and Pensions, 2014), and the fact that both disability and cancer incidence increase with age. We found that participation in screening varied by type of disability and number of disabilities. There were greater disparities by disability status for breast screening than for bowel screening. In women with a disability, not having access to a car was associated with a further reduction in the likelihood of participating in breast screening, as it does for women in general (Moser *et al*, 2009), presumably because of the extra effort required to go to breast screening centres. These results provide the NHS screening programmes with objective evidence of inequity and the results may assist in the development of future policy.

Previous evidence from the United States has suggested that women with disabilities are less likely to attend breast screening (Chevarley *et al*, 2006; Courtney-Long *et al*, 2011; Horner-Johnson *et al*, 2014). Until now, however, what little evidence was available from the United Kingdom suggested no corresponding reduction in uptake of screening for people with disabilities (Graham *et al*, 1998; Solmi *et al*, 2015). See Supplementary File p5 for further references.

A wide range of potential barriers to screening for women with disabilities have been identified. In the case of breast screening, these barriers include problems with transport to, and physical access to, screening centres, positioning during mammography, communication problems with staff, and perceived attitudes of staff (Llewellyn *et al*, 2011, Angus *et al*, 2012, Peters and Cotton, 2015). In the case of bowel screening, an evaluation of the NHS screening pilot reported that physical disability was cited by participants as a reason for non-completion of the test (Alexander and Weller, 2003).

Strengths of this study are the prospective design with the recording of disability prior to follow-up for screening and the use of an objective measure for screening participation. This avoids any bias from retrospective reporting of disability or over-reporting of participation in screening by self-reports. The study sample may not be fully representative of the population in England who are eligible for screening because the Million Women Study participants were originally recruited through the NHS breast screening programme and have therefore previously attended for breast screening at least once. They are also more likely to participate in bowel screening (77% in this study *vs* 50% in England as a whole; Logan *et al*, 2012). In addition, the study sample is not fully representative of the original Million Women Study cohort as responders to the 8-year re-survey tended to be less deprived, more educated, and to be less likely to smoke than non-responders. However, none of the above should bias comparisons within the cohort.

In conclusion, women in England with disabilities, especially in domains of self-care, vision, and mobility, are considerably less likely than other women to accept invitations to participate in routine screening for breast or bowel cancer.

## The Million Women Study Collaborators

The Million Women Study Advisory Committee are: Emily Banks, Valerie Beral, Lucy Carpenter, Carol Dezateux, Jane Green, Julietta Patnick, Richard Peto, Cathie Sudlow. The co-ordinating staff for the Million Women Study are: Hayley Abbiss, Simon Abbott, Krys Baker, Angela Balkwill, Isobel Barnes, Valerie Beral, Judith Black, Kathryn Bradbury, Anna Brown, Benjamin Cairns, Dexter Canoy, Andrew Chadwick, Dave Ewart, Sarah Ewart, Sarah Floud, Toral Gathani, Laura Gerrard, Adrian Goodill, Jane Green, Lynden Guiver, Darren Hogg, Alicia Heath, Carol Herman, Isobel Lingard, Sau Wan Kan, Nicky Langston, Kirstin Pirie, Gillian Reeves, Keith Shaw, Emma Sherman, Rachel Simpson, Helena Strange, Sian Sweetland, Sarah Tipper, Anthony Webster, Claire Wotton, Lucy Wright, Owen Yang, and Heather Young. The following NHS breast screening centres took part in the recruitment and breast screening follow-up for the Million Women Study: Avon, Aylesbury, Barnsley, Basingstoke, Bedfordshire & Hertfordshire, Cambridge & Huntingdon, Chelmsford & Colchester, Chester, Cornwall, Crewe, Cumbria, Doncaster, Dorset, East Berkshire, East Cheshire, East Devon, East of Scotland, East Suffolk, East Sussex, Gateshead, Gloucestershire, Great Yarmouth, Hereford & Worcester, Kent (Canterbury, Rochester, Maidstone), Kings Lynn, Leicestershire, Liverpool, Manchester, Milton Keynes, Newcastle, North Birmingham, North East Scotland, North Lancashire, North Middlesex, North Nottingham, North of Scotland, North Tees, North Yorkshire, Nottingham, Oxford, Portsmouth, Rotherham, Sheffield, Shropshire, Somerset, South Birmingham, South East Scotland, South East Staffordshire, South Derbyshire, South Essex, South Lancashire, South West Scotland, Surrey, Warrington Halton St Helens & Knowsley, Warwickshire Solihull & Coventry, West Berkshire, West Devon, West London, West Suffolk, West Sussex, Wiltshire, Winchester, Wirral and Wycombe.

## Figures and Tables

**Table 1:** Characteristics of participants invited for either breast or bowel cancer screening by disability status

	**Any disability**^a^ (***N*****=109 869)**	**No disability (*****N*****=363 316)**
	***n*** **(%)**	
Age ⩾65 years at re-survey	40 092 (36)	104 027 (29)
White ethnicity	86 520 (99)	306 605 (99)
Most deprived quintile	28 294 (26)	48 921 (14)
No educational qualifications^b^	50 084 (47)	110 095 (31)
Not married or living with a partner	28 711 (27)	70 161 (20)
No access to a car	17 421 (16)	27 040 (8)
Body mass index ⩾30 kg m^−2^	37 467 (37)	49 134 (14)
Current smoker	13 591 (13)	29 012 (8)

a Includes each type of disability and receipt of disability benefits.

b No qualifications combines two categories: those who completed compulsory schooling and those who did not.

**Table 2:** Relative risks (95% CIs) for participating in cancer screening for women with any disability versus no disability within certain subgroups

	**Breast cancer screening**	**Bowel cancer screening**
	**RR (95% CI)**	
Access to a car	0.65 (0.63–0.67)	0.75 (0.74–0.77)
No access to a car	0.58 (0.54–0.61)	0.72 (0.69–0.75)
*P* for heterogeneity	0.001	0.034
Married/living with a partner	0.64 (0.62–0.66)	0.74 (0.73–0.76)
Not married/living with a partner	0.60 (0.57–0.63)	0.72 (0.69–0.74)
*P* for heterogeneity	0.038	0.056

Abbreviations: CI=confidence interval; RR=relative risk. Note: any disability includes any type of disability and receipt of disability benefits.

RRs were adjusted for age, region, deprivation, ethnicity, education, body mass index, and smoking, and by marital status and car availability as appropriate.
