# Supplementary material for: Disability and participation in breast and bowel cancer screening in England: a large prospective study
Source: Br J Cancer. 2017 Oct 3;117(11):1711–4. doi: 10.1038/bjc.2017.331 (PMC5729433; doi:10.1038/bjc.2017.331)
Supplement: Supplementary Material [file bjc2017331x1.docx]

**Supplementary File**

**Disability and participation in breast and bowel cancer screening in England: a large prospective study**

S Floud*^1^, I Barnes^1^, M Verfürden^2^, H Kuper^3^, T Gathani^14^, R G Blanks^1^, R Alison^1^, J Patnick^1^, V Beral^1^, J Green^1^, G K Reeves^1^, for the Million Women Study Collaborators

*Corresponding author

[sarah.floud@ceu.ox.ac.uk](mailto:sarah.floud@ceu.ox.ac.uk)

^1^ Cancer Epidemiology Unit, Nuffield Department of Population Health, University of Oxford, Roosevelt Drive, Oxford, OX3 7LF, UK

^2^UCL Great Ormond Street Institute of Child Health, 30 Guilford Street, London, WC1N 1EH, UK

^3^International Centre for Evidence in Disability, London School of Hygiene & Tropical Medicine, Keppel Street, London WC1E 7HT, UK

^4^Oxford University Hospitals NHS Foundation Trust, John Radcliffe Hospital, Headley Way, Headington, Oxford OX3 9DU

Contents

[Table S1 2](#_Toc486426178)

[Table S2 3](#_Toc486426179)

[Figure S1 4](#_Toc486426180)

[Further references 5](#_Toc486426181)

# Table S1

| **Table S1: Disabilities reported by study participants invited for screening** | | |
| --- | --- | --- |
|  | **Women invited for breast cancer screening** | **Women invited for bowel cancer screening** |
|  | ***N* = 445,579** | ***N* = 449,058** |
|  | **% (n)** | **% (n)** |
| No disability | 77.2 (343,952) | 77.1 (346,041) |
| Any disability† | 22.8 (101,627) | 22.9 (103,017) |
| Number of disabilities: |  |  |
| 1 | 15.2 (67,789) | 15.3 (68,707) |
| 2 | 5.7 (25,310) | 5.7 (25,646) |
| 3+ | 1.2 (5,193) | 1.2 (5,271) |
| Type of disability: |  |  |
| Hearing | 3.3 (14,555) | 3.3 (14,864) |
| Memory | 2.4 (10,535) | 2.4 (10,610) |
| Mobility | 17.8 (79,121) | 17.9 (80,245) |
| Vision | 1.5 (6,493) | 1.5 (6,552) |
| Self-care | 5.4 (24,253) | 5.5 (24,492) |
| Receipt of disability benefits | 8.0 (35,422) | 8.0 (35,972) |
| †includes each type of disability and receipt of disability benefits | | |

# Table S2

| **Table S2 Characteristics of participants invited for either breast or bowel cancer screening by type of disability** | | | | | |
| --- | --- | --- | --- | --- | --- |
|  | **Type of disability** | | | | |
|  | **Hearing**  **(*N* = 15,797)** | **Memory**  **(*N* = 11,202)** | **Mobility**  **(*N* = 85,834)** | **Vision**  **(*N* = 6,960)** | **Self-care**  **(*N* = 26,169)** |
|  | **n (%)** | **n (%)** | **n (%)** | **n (%)** | **n (%)** |
| ≥ 65 years old at re-survey | 5,960 (38) | 3,281 (29) | 32,212 (38) | 2,373 (34) | 9,320 (36) |
| White ethnicity | 12,577 (99) | 8,614 (98) | 66,965 (98) | 5,290 (98) | 20,129 (99) |
| Most deprived quintile | 3,793 (24) | 3,046 (27) | 23,512 (28) | 2,187 (32) | 8,342 (32) |
| No educational qualifications‡ | 7,199 (47) | 5,439 (50) | 40,460 (48) | 3,348 (50) | 13,216 (52) |
| Not married or living with a partner | 3,851 (25) | 3,097 (29) | 23,154 (28) | 1,914 (28) | 7,622 (30) |
| No access to a car | 2,364 (15) | 1,896 (17) | 14,283 (17) | 1,718 (25) | 4,969 (20) |
| Body mass index ≥30 kg/m^2^ | 3,635 (25) | 2,793 (27) | 33,516 (43) | 1,782 (28) | 10,504 (44) |
| Current smoker | 1,824 (12) | 1,484 (13) | 11,031 (13) | 1,115 (16) | 3,776 (15) |
| ‡no qualifications combines two categories: those who completed compulsory schooling and those who did not | | | | | |

# Figure S1

**Relative risks (95% CIs) for participating in breast and bowel cancer screening by various measures of disability, restricted to women with complete information on all variables.**

Any disability includes any type of disability and receipt of disability benefits. Relative risks are adjusted for age, region, deprivation, ethnicity, marital status, car availability, body mass index and smoking.


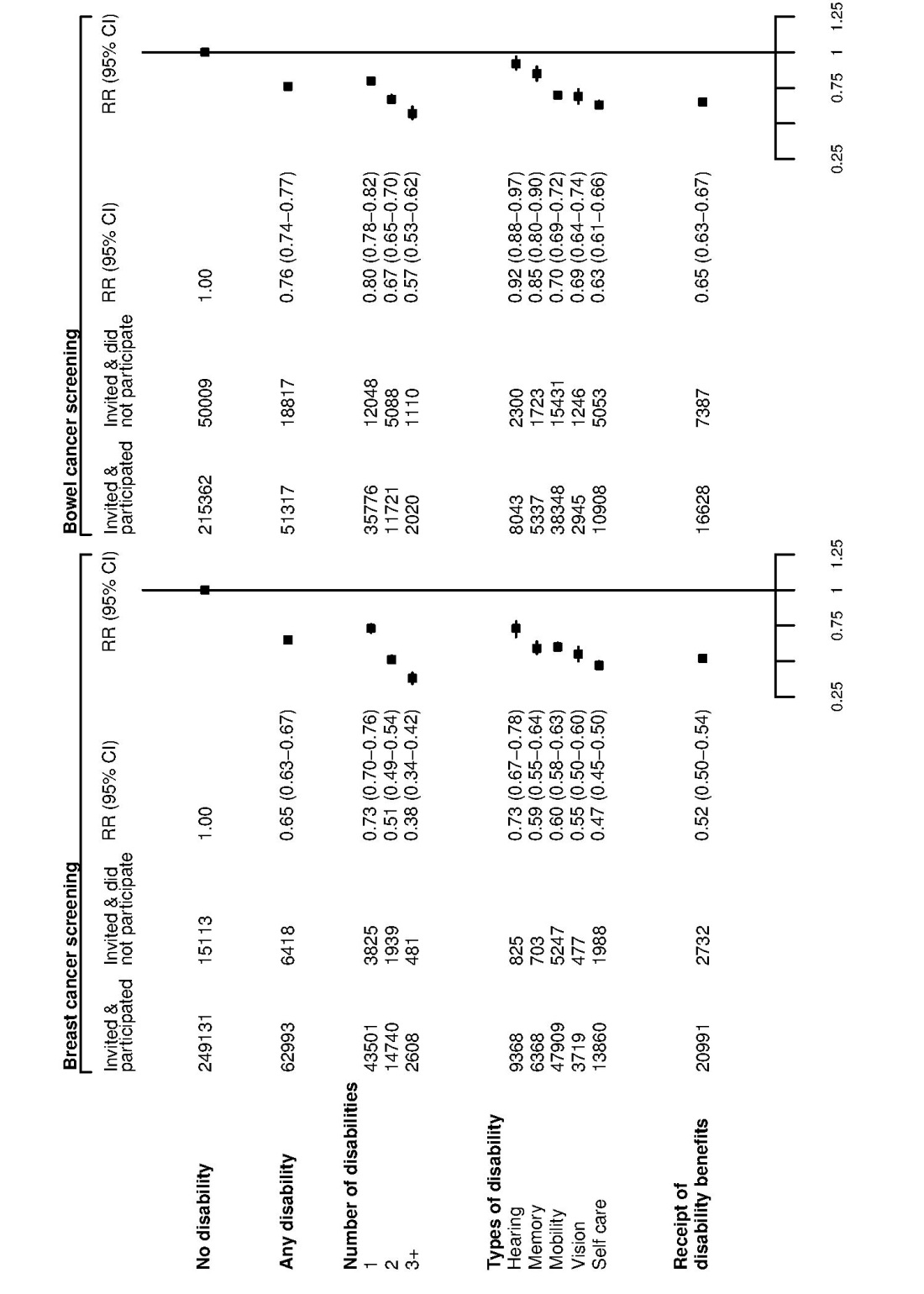


# Further references

Bussiere, C., Sicsic, J. & Pelletier-Fleury, N. 2014. The effects of obesity and mobility disability in access to breast and cervical cancer screening in france: results from the national health and disability survey. *PLoS One,* 9.

Guilcher, S. J., Lofters, A., Glazier, R. H., Jaglal, S. B., Voth, J. & Bayoumi, A. M. 2014. Level of disability, multi-morbidity and breast cancer screening: does severity matter? *Prev Med,* 67**,** 193-198.

Iezzoni, L. I., McCarthy, E. P., Davis, R. B., Harris-David, L. & O'Day, B. 2001. Use of screening and preventive services among women with disabilities. *Am J Med Qual,* 16**,** 135-144.

Iezzoni, L. I., McCarthy, E. P., Davis, R. B. & Siebens, H. 2000. Mobility impairments and use of screening and preventive services. *Am J Public Health,* 90**,** 955-961.

Kinnear, H., Rosato, M., Mairs, A., Hall, C. & O'Reilly, D. 2011. The low uptake of breast screening in cities is a major public health issue and may be due to organisational factors: A Census-based record linkage study. *Breast,* 20**,** 460-463.

Mele, N., Archer, J. & Pusch, B. D. 2005. Access to breast cancer screening services for women with disabilities. *J Obstet Gynecol Neonatal Nurs,* 34**,** 453-464.

Power, E., Miles, A., von Wagner, C., Robb, K. & Wardle, J. 2009. Uptake of colorectal cancer screening: system, provider and individual factors and strategies to improve participation. *Future Oncol,* 5**,** 1371-1388.

van Jaarsveld, C. H., Miles, A., Edwards, R. & Wardle, J. 2006. Marriage and cancer prevention: does marital status and inviting both spouses together influence colorectal cancer screening participation? *J Med Screen,* 13**,** 172-176.

Wei, W., Findley, P. A. & Sambamoorthi, U. 2006. Disability and receipt of clinical preventive services among women. *Womens Health Issues,* 16**,** 286-296.

Williams, J., Garvican, L., Tosteson, A. N., Goodman, D. C. & Onega, T. 2015. Breast cancer screening in England and the United States: a comparison of provision and utilisation. *Int J Public Health,* 60**,** 881-890.
